# Metformin versus insulin in gestational diabetes mellitus: a systematic review

**DOI:** 10.61622/rbgo/2024rbgo89

**Published:** 2024-12-04

**Authors:** Giovanna Noronha Berti, Igor Gutschov Oviedo Garcia, João Pedro Ruas Floriano de Toledo, Júlia Rodrigues Tatemoto, Lais Watanabe Marino, Mariana de Medeiros Legori, Sérgio Floriano de Toledo

**Affiliations:** 1 Centro Universitário Lusíada Santos SP Brazil Centro Universitário Lusíada, Santos, SP, Brazil.

**Keywords:** Gestational diabetes, Insulin therapy, Metformin, Pregnancy complications

## Abstract

**Objective:**

The aim of this study is to assess the use of metformin with or without insulin for the treatment of Gestational Diabetes Mellitus compared to insulin alone.

**Data sources:**

This article consists of a systematic review of randomized clinical trials. The searches were carried out on MEDLINE including 7 studies, between 2010 to 2021.

**Study selection:**

Randomized clinical trials comparing metformin and insulin written in English, Spanish or Portuguese, with no time limit, were included.

**Data collection:**

Data was extracted from all the 7 articles and compared statistically when possible. Whenever data was not available or couldn't be statistically compared, the main results were described in detail.

**Data synthesis:**

Insulin alone is not superior than metformin with or without insulin on gestational diabetes mellitus.

**Conclusion:**

There is a potential viability of using metformin as an alternative compared to insulin alone in the treatment of Gestational Diabetes Mellitus. However, all assessed outcomes have a very low level of certainty of evidence and more studies are necessary to support these findings.

## Introduction

Gestational Diabetes Mellitus (GDM) is the most prevalent clinical complication in the pregnancy-postpartum cycle, representing a significant public health concern, with a substantial increase in its prevalence over the past decades. GDM affects approximately 16.5% of pregnancies worldwide, and this number is expected to rise with the increasing obesity epidemic. The combination of diabetes and pregnancy without adequate metabolic control can be associated with adverse maternal, fetal, and neonatal outcomes, such as the possibility of macrosomia, neonatal hypoglycemia, prematurity, hyperbilirubinemia, and fetal birth injuries. In an effort to prevent adverse maternal, fetal, and neonatal outcomes, the initial therapeutic approach for GDM includes nutritional guidance and regular physical activity. However, some pregnant women are unable to manage this disorder solely through lifestyle changes, necessitating the use of medications. Insulin is currently considered the gold standard for achieving euglycemia in pregnant women. Metformin, a first-line medication for the treatment of Type 2 Diabetes Mellitus, can be administered in specific situations, such as when obtaining a glucometer or insulin is impossible, when a pregnant woman is already using 100 international units (IU) of insulin and metformin needs to be added, and when she is unable to correctly use insulin. This drug has been recognized as a viable alternative to insulin therapy for GDM.^(1–6)^

Metformin is a biguanide that reduces gluconeogenesis in the liver and stimulates glucose uptake in peripheral tissues.^(7)^ It reduces both fasting and postprandial plasma glucose. Besides, it does not induce hypoglycemia and it is not associated with increased weight gain.^(8)^ Different from insulin which requires an insulin-antibody complex to cross the placental barrier, metformin can freely traverse the placenta and circulate in the foetus.^(9,10)^ Despite that, there is no evidence of adverse fetal effects or increased risk of major malformations when metformin is used in pregnant women.^(8)^ Patients initiating metformin treatment may experiencing mild gastrointestinal adverse effects. These may encompass diarrhea, abdominal discomfort, loss of appetite, nausea, and occasionally, a metallic taste in the mouth. These symptoms tend to correlate with the dosage and typically improve if the dosage is decreased.^(11)^

Metformin is contraindicated when eGFR is less than 30 mL/min. Moreover, metformin prescription should be carefully done in patients with acute heart failure, especially when there is hypoperfusion and hypoxemia. Metformin seems to be safe for patients with stable compensated heart failure and sufficient end-organ perfusion. The FDA label also expresses worries about metformin-triggered lactic acidosis in liver failure, potentially due to hindered lactate clearance.^(12)^ There is still debate regarding the therapeutic equivalence between metformin and insulin in the treatment of gestational diabetes mellitus. Hence, the need arose to conduct a Systematic Review on the topic to consolidate the benefits and drawbacks, assessing both maternal and neonatal outcomes. To achieve this, we defined the primary outcomes for study as: birth weight, mode of delivery, need for neonatal intensive care unit (NICU) admission, gestational age at birth, neonatal hypoglycemia, neonates large for gestational age at birth, progression to preeclampsia, and Apgar score at 5 minutes of life. Therefore, we conducted a Systematic Review using data from Randomized Clinical Trials, aiming to provide more evidence regarding the efficacy and safety of insulin and metformin, and to compare the benefits and drawbacks of these two therapies.

## Methods

This article constitutes a systematic review of the available literature. The selection of articles was conducted through a search in the MEDLINE (PUBMED) database, with no time limit. The review process was carried out following the Preferred Reporting Items for Systematic Review and Meta-Analysis guidelines.^(13)^ Only randomized clinical trials were selected for inclusion. The following outcomes were investigated: birth weight, mode of delivery, need for NICU admission, gestational age at birth, neonatal hypoglycemia, neonates large for gestational age at birth, progression to preeclampsia, and Apgar score at 5 minutes of life.

## Eligibility criteria for studies

### Inclusion criteria

Randomized clinical trials, full-text articles available for reading and data extraction, patients with current gestational diabetes mellitus; comparison between two groups of patients, with one group using metformin and the other insulin therapy, and studies that present at least one of the following outcomes: birth weight, mode of delivery, need for NICU admission, gestational age at birth, neonatal hypoglycemia, neonates large for gestational age at birth, progression to preeclampsia, and Apgar score at 5 minutes of life.

### Exclusion criteria

Study design other than a randomized clinical trial; studies without a comparison between the chosen medications; systematic reviews; guidelines; studies in languages other than English, Spanish, and Portuguese; articles unavailable in full text; and articles that did not assess the selected outcomes.

## Search strategy

### Structured question

We utilized the following components of the structured question under the acronym PICO (Population, Intervention, Comparison, Outcome) to guide the database search and selection of studies:

- P: Patients with gestational diabetes mellitus.
- I: Insulin therapy.
- C: Metformin.
- O: Birth weight; mode of delivery; need for NICU admission; gestational age at birth; neonatal hypoglycemia; neonates large for gestational age at birth; progression to preeclampsia; Apgar score at 5 minutes of life.

The search strategy conducted in the database used the following descriptors: (Pregnancy OR Pregnancies OR Gestation OR Gestations OR Gravidity OR Gravidities OR Parity OR Parities OR Parturition OR Parturitions) AND (Diabetes Pregnancy-Induced OR Diabetes Pregnancy Induced OR Pregnancy-Induced Diabetes OR Gestational Diabetes OR Diabetes Mellitus Gestational OR Gestational Diabetes Mellitus OR Diabetes Gestational) AND (Isophane Insulin OR Insulin OR Neutral Protamine Hagedorn Insulin OR Protamine Hagedorn Insulin OR Hagedorn Insulin Protamine OR Insulin NPH OR NPH Insulin OR Insulin Protamine Zinc OR Protamine Zinc Insulin OR Zinc Insulin Protamine) AND (Metformin OR Dimethylbiguanidine OR Dimethylguanylguanidine OR Glucophage OR Metformin Hydrochloride OR Hydrochloride Metformin OR Metformin HCl OR HCl Metformin).

### Data collection process

Seven authors independently assessed, based on predefined inclusion and exclusion criteria, the retrieved article titles, abstracts, and, finally, the full texts. The process of including and excluding studies was documented in a flow diagram following the PRISMA recommendations and presented in the results. Data was extracted for analysis into an Excel table (Microsoft Office 2017) containing: study identification (author, year of publication, study design) and data on the assessed outcomes; conclusions from individual articles.

### Risk of bias assessment for included studies

The assessment of the risk of bias for the included studies was conducted individually and independently by all seven authors, using the "Cochrane Risk of Bias" tool (RoB 2.0). The following domains were evaluated:

1. Randomization of the sample and blinded allocation
2. Blinding of participants and researchers
3. Loss of data
4. Outcome analysis
5. Selection of reported outcomes

We also noted whether the included articles reported any sample size and power calculations, as well as articles that ended prematurely due to futility or other reasons. Discrepancies were resolved through consensus or by the decision of the seventh researcher (Garcia IGO). The results of the bias risk analysis are presented in the Bias Risk tables (characteristics of included studies).

### Measures of effects

For dichotomous data, we calculated the results for each study individually in the form of risk difference and used a 95% confidence interval (CI). We also calculated the relative risk (RR) with a 95% CI to fully report the certainty of the evidence later. For continuous data, we used the mean difference in each study and standard deviation. We used the Review Manager (RevMan) 5.4 software for the statistical calculations.

### Data synthesis

We used a random-effects model for eligible studies using Review Manager (RevMan) 5.4 software to perform calculations and generate Forest Plot graphs for each outcome. For outcomes where quantitative synthesis was not feasible, we conducted a qualitative analysis of the results obtained from the studies, presenting them in tables and discussing them throughout the text.

### Certainty of evidence

We used the GRADE approach (The Grading of Recommendations Assessment, Development and Evaluation Approach) to assess the certainty of evidence for each outcome based on: risk of bias in the studies; unexplained heterogeneity or inconsistency; presence of indirect evidence; imprecision in the results; high risk of publication bias. We decreased the certainty level of the evidence by one if the risk was serious and by two if the risk was very serious. The results of the comparisons were discussed in the discussion section of the paper.^(14)^

## Results

### Study selection

**Figure 1:**
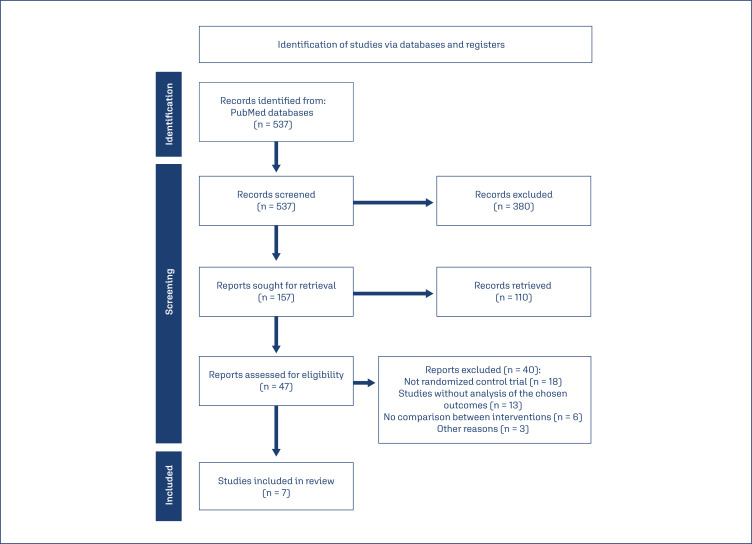
PRISMA flowchart

The search conducted in July 2023 retrieved 537 articles, of which 157 were selected based on the title. Among these, 47 articles were chosen after reading the abstract. Forty articles were excluded from the present study for the following reasons: 18 did not match the proposed study design (randomized clinical trial), 13 did not report the chosen outcomes, and 6 did not compare the interventions (metformin and insulin). Three articles were excluded for other reasons (foreign language other than English or Spanish; study conducted with pregnant women with pre-gestational diabetes; study not completed). In accordance with the eligibility criteria, seven articles were included, as depicted in the PRISMA flowchart shown in figure 1. Due to the heterogeneity of the studies, it was not possible to conduct a quantitative synthesis, and only a qualitative synthesis was performed.^(13)^

### Studies characteristics

The characteristics of the included studies are described in chart 1.

**Chart 1:** Characteristics of included studies

Author	Study	Number of participants	Insulin (UI/kg/day)	Metformin (mg/day)	BMI intervention (kg/m²)*	BMI comparison (kg/m²)*	Age intervention (years)*	Age comparison (years)*
Ijäs et al. (2010)^(15)^	Randomized Clinical Trial	100	30 UI/day (average)	750 – 2250	30,8 ± 5,4	31,5 ± 6,5	31,7 ± 6,1	32,3 ± 5,6
Spaulonci et al. (2013)^(16)^	Randomized Clinical Trial	92	0,4	1700 – 2550	32,04 ± 4,7	31,99 ± 4,92	32,76 ± 4,66	31,93 ± 6,02
Ruholamin et al. (2014)^(17)^	Randomized Clinical Trial	119	0,2	500 – 1500	25,1 ± 3,4	26,4 ± 2,8	23,4 ± 2,5	24,6 ± 6,3
Ainuddin et al. (2015)^(18)^	Randomized Clinical Trial	150	0,9	500 – 2500	N/A	N/A	31 ± 4	30,6 ± 2,9
Simeonova-Krstevska et al. (2018^)(19^)	Randomized Clinical Trial	349	0,3	500 – 2000	27,5 ± 4,9	28,8 ± 5,3	32,7 ± 5,7	32,2 ± 4,7
Ghomian et al. (2019^)(20^)	Randomized Clinical Trial	286	0,1	500 – 1500	24,0 ± 2,10	23,73 ± 1,87	28,41 ± 6,36	28,30 ± 5,25
Picón-César et al. (2021)^(8)^	Randomized Clinical Trial	200	0,1 – 0,5	425 – 2550	30,42 ± 5,42	29,89 ± 5,73	37,86 ± 4,83	34,81 ± 5,24

* data on average; ± standard deviation

### Risk of bias assessment

**Figure 2:**
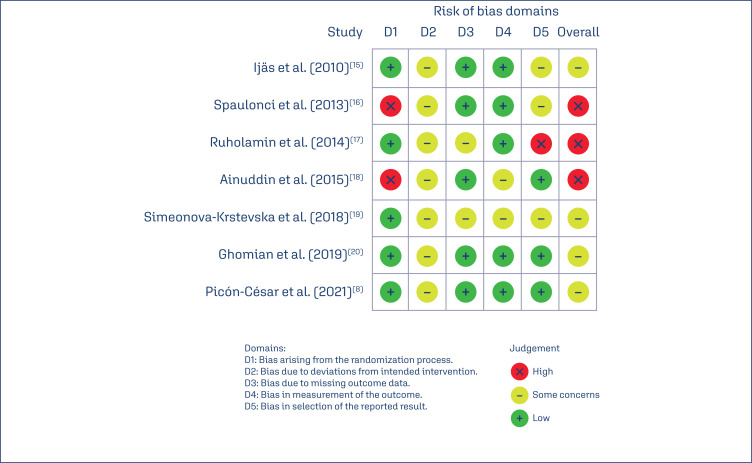
RoB-2.0 tool

We applied the RoB-2.0 tool to assess the risk of bias in the included studies, with results as depicted in the figures. None of the studies had a low risk of overall bias, with four of them having a moderate risk of bias and three of them having a high risk of bias. The main issues found were related to bias in the randomization and blinding process (2/7) and bias due to deviations from the intended intervention (7/7), as it is not possible to blind patients and researchers due to the form of drug administration (Figure 2).

### Individual results of studies

Studies had significant heterogeneity mainly due to the lack of standardization in the administration of the drugs studied. Therefore, for the synthesis of evidence, we separated the studies into subgroups according to the dose used for both metformin and insulin, as shown in the following chart 2.

**Chart 2:** Individual results of studies

	Maternal outcomes			Neonatal outcomes				
Author	Progression to pre-eclampsia^1^	Gestational age at birth (weeks)^2^	Cesarean delivery^1^	Birth Weight (grams)^2^	Admission to neonatal ICU^1^	LGA at Birth^1^	Occurrence of neonatal hypoglycemia^1^	APGAR in the 5^th^ minute^3^
Ijäs et al. (2010)^(15)^	-0,01 [-0,11, 0,10]	0,40 [-0,2, 1,00]	-0,18 [-0,36, -0,01]	-154 (-359,60, 51,60)	0,07 [-0,08, 0,22]	0,01 [-0,10, 0,13]	-0,05 [-0,20, 0,10]	8,9 ± 0,8
Spaulonci et al. (2013)^(16)^	-0,07 [-0,22, 0,09]	-0,09 [-0,70, 0,52]	-0,07 [-0,25, 0,12]	94 (-118,81, 306,81)	–	0,07 [-0,02, 0,15]	0,07 [-0,09, 0,22]	10
Ruholamin et al. (2014)^(17)^	0,02 [-0,07, 0,11]	–	-0,04 [-0,22, 0,14]	166 (-19,5, 351,5)	0,04 [-0,03, 0,11]	0,00 [-0,04, 0,04]	0,04 [-0,03, 0,11]	Apgar 7 - 1 Apgar 8 - 16 Apgar 9 - 33
Ainuddin et al. (2015)^(18)^	0,08 [0,01, 0,15]	0,40 [-0,13, 0,93]	0,09 [-0,10, 0,27]	300 (135,38, 464,62)	0,19 [0,03, 0,36]	0,14 [-0,03, 0,22]	0,17 [0,05, 0,28]	8,6 ± 0,9
Simeonova-Krstevska et al. (2018)^(19)^	-0,02 [-0,11, 0,07]	-1,40 [-1,98, -0,82]	0,15 [-0,02, 0,32]	-148 (-346,02, 50,02)	–	0,09 [-0,03, 0,22]	0,16 [-0,01, 0,33]	8,6 ± 0,7
Ghomian et al. (2019)^(20)^	–	–	0,06 [-0,05, 0,18]	94 (3,70, 184,30)	-0,01 [-0,11, 0,08]	–	0,03 [-0,03, 0,10]	Apgar <7 - 16 Apgar ≥ 7 - 127
Picón-César et al. (2021)^(8)^	-0,01 [-0,04, 0,02]	0,02 [-0,51, 0,55]	0,25 [0,11, 0,38]	62 (-94,40, 218,40)	0,00 [-0,02, 0,02]	0,05 [-0,06, 0,15]	0,15 [0,06, 0,24]	9,77 ± 0,55

RD: Risk difference; MD: Mean Difference; CI95%: Confidence Interval 95%;

1 Risk difference (CI95%);

2 Mean difference (CI95%);

3 Mean; ± standard deviation or number of neonates with each score

### Grade profile and summary of findings

We used the GRADE tool to assess the certainty of evidence for the analyzed outcomes as per Appendix A. The summaries of findings are presented in the chart 3.^(14)^

**Chart 3:** Summary of findings

Metformin compared to Insulin therapy for Diabetes Mellitus Gestacional						
Patient or population: Gestational Diabetes Mellitus Setting: Pregnant women diagnosed with gestational diabetes mellitus Intervention: Metformin Comparison: Insulin therapy						
Outcome and № of participants (studies)	Relative effect (95% CI)	Anticipated absolute effects			Certainty	What happens
		Events involving insulin therapy	Events involving Metformin	Risks difference (95% CI)/ Means difference		
Large for Gestational Age at birth № of participants: 752 (6 RCTs)	RR ranged from 1,18 (0,34, 4,11) to 7 (0,37, 131,81)	77/421	34/331	MD ranged from 0 (-0,04, 0,04) to 0,14 (-0,03, 0,31)	⨁◯◯◯ Very low^a,b,c^	In pregnant women using metformin, newborns large for gestational age at birth were less frequent than in those using insulin therapy only, however, the evidence is very uncertain.
Progression with pre-eclampsia № of participants: 752 (6 RCTs)	RR ranged from 0.33 (0,01, 7,92) to 7.53 (0,43, 130,42)	26/421	21/331	MD ranged from -0,07 (-0,22, 0,09) to 0,08 (0,01, 0,15)	⨁◯◯◯ Very low^a,b,d^	Metformin may have little or no effect on the occurrence of preeclampsia in pregnant women with gestational diabetes mellitus, however, the evidence is very uncertain.
Need for admission to the neonatal intensive care unit № of participants: 797 (5 RCTs)	RR ranged from 0.93 (0,58, 1,49) to 5.00 (0,25, 101,58)	72/417	46/380	MD ranged from -0,01 (-0,11, 0,08) to 0,19 (0,03, 0,36)	⨁◯◯◯ Very low^b,d,e^	Metformin may have little or no effect on the need for admission to the neonatal intensive care unit, however, the evidence is very uncertain.
Cesarean delivery № of participants: 1038 (7 RCTs)	RR ranged from 0.52 (0,27, 1,01) to 1.92 (1,31, 2,81)	295/564	236/474	MD ranged from -0,18 (-0,36, -0,01) to 0,25 (0,11, 0,38)	⨁◯◯◯ Very low^b,d,f^	Metformin may have little or no effect on the need for cesarean delivery, however, the evidence is very uncertain.
Neonatal hypoglycemia № of participants: 1038 (7 RCTs)	RR ranged from 0.73 (0,30, 1,81) to 5.00 (0,25, 101,58)	121/564	53/474	MD ranged from -0,05 (-0,20, 0,10) to 0,17 (0,05, 0,28)	⨁◯◯◯ Very low^b,d,f^	Metformin may have little or no effect on the occurrence of neonatal hypoglycemia, however, the evidence is very uncertain
Birth weight № of participants: 1038 (7 RCTs)	–	The mean birth weight ranged from 3233 to 3700 grams	-	MD ranged from -154 to 300 grams	⨁◯◯◯ Very low^b,f,g^	
Gestational age at birth № of participants: 652 (5 RCTs)	-	The mean Gestacional age at birth ranged from 37,5 39,3 weeks	-	MD ranged from -1,4 to 0,4 weeks	⨁◯◯◯ Very low^b,d,e^	
*The risk in the intervention group (and its 95% confidence interval) is based on the assumed risk in the comparison group and the relative effect of the intervention (and its 95% CI). CI: confidence interval; MD: mean difference; RR: risk ratio						
GRADE Working Group grades of evidence High certainty: we are very confident that the true effect lies close to that of the estimate of the effect. Moderate certainty: we are moderately confident in the effect estimate: the true effect is likely to be close to the estimate of the effect, but there is a possibility that it is substantially different. Low certainty: our confidence in the effect estimate is limited: the true effect may be substantially different from the estimate of the effect. Very low certainty: we have very little confidence in the effect estimate: the true effect is likely to be substantially different from the estimate of effect.						

## Discussion

Regarding the diagnostic criteria for defining GDM, the selected articles in the study were based on 5 different guidelines. Spaulonci et al.,^(16)^ Ainuddin et al.,^(18)^ and Ghomian et al.^(20)^ used the guidelines of the American Diabetes Association (ADA), which are based on the values of the 75g oral glucose tolerance test (OGTT). At the time the cited studies were conducted, the diagnosis was established when the patient presented with 2 or more of the following altered values: > 95 mg/dL (fasting), ≥ 180 mg/dL (1 hour), ≥ 155 mg/dL (2 hours), and ≥ 140 mg/dL (3 hours).^(21)^

Ruholamin et al.^(17)^ used the Australasian Diabetes in Pregnancy Society diagnostic criteria, which diagnose) GDM when one or more of the following glucose levels are elevated on the 75 g OGTT: ≥ 5.1 mmol/L (fasting), ≥ 10.0 mmol/L (1 hour), and ≥ 8.5 mmol/L (2 hours).^(22)^

Picón-César et al.^(8)^ used the National Diabetes Data Group (NDDG) criteria. Firstly, a 50 g oral glucose challenge test (GCT) is performed as a screening test. If the GCT is positive (≥ 140 mg/dL), then the pacient needs to undergo the 100g OGTT for diagnostic confirmation. The diagnosis is established when one or more of the following values is present: ≥ 105 mg/dL (fasting), ≥ 190 mg/dL (1 hour), ≥ 165 mg/dL (2 hours), and ≥ 145 mg/dL (3 hours).^(23)^

Simeonova-Krstevska et al.^(19)^ used the diagnosis for GDM according to The International Association for Diabetes and Pregnancy Study Group (IADPSG). The diagnosis can be made during the first prenatal visit checking Fasting Plasma Glucose (FPG): If FPG is ≥ 5.1 mmol/l (92 mg/dl) but < 7.0 mmol/l (126 mg/dl), diagnose as GDM. If FPG is < 5.1 mmol/l (92 mg/dl), conduct a 75-gram Oral Glucose Tolerance Test (OGTT) between 24 to 28 weeks’ gestation to screen for GDM. Subsequently, at 24–28 weeks of gestation, the diagnosis of GDM is based on the Fasting Plasma Glucose (FPG): ≥ 5.1 mmol/l (92 mg/dl), including Cumulative proportion of HAPO cohort equaling or exceeding this threshold: 8.3%. Later, 1-hour Plasma Glucose during Oral Glucose Tolerance Test (OGTT) consider: ≥ 10.0 mmol/l (180 mg/dl), including Cumulative proportion of HAPO cohort equaling or exceeding this threshold: 14.0%. Lastly, 2-hour Plasma Glucose during Oral Glucose Tolerance Test (OGTT): ≥ 8.5 mmol/l (153 mg/dl).^(24)^

Regarding the birth weight outcome, the articles by de Picón-César et al.,^(8)^ Ijäs et al.,^(15)^ Spaulonci et al.,^(16)^ Ruholamin et al.,^(17)^ and Simeonova-Krstevska et al.^(19)^ did not present results with statistically significant differences, while the works of Ainuddin et al.^(18)^ and Ghomian et al.^(20)^ did. Both studies showed that there was less weight gain in neonates born to mothers who used metformin alone as an antidiabetic. This result is consistent with what was observed in the systematic review and meta-analysis conducted by Sheng et al.,^(25)^ which analyzed 22 articles involving 4174 neonates, indicating a significantly lower birth weight in the children of patients undergoing metformin therapy (mean difference 122.76, 95% CI -178.31, -67.21; I^2^ = 84%; p < 0.0001).^(15–20,25)^

In summary, although there is no clear evidence that metformin presents a lower risk of excessive weight gain, it is observed that in both groups, the mean birth weight remained below the threshold for the diagnosis of macrosomia.

When evaluating the cesarean delivery outcome, it was observed that two articles showed statistically significant differences: Picón-César et al.,^(8)^ and Ijäs et al.^(15)^ Regarding the study by Ijäs et al.,^(15)^ a risk difference of -0.18 (95% CI -0.36, -0.01) in favor of insulin use to prevent cesarean delivery was observed, with the majority being performed vaginally.^(8,15)^

However, Picón-César et al.^(8)^ showed the opposite: 51 cesarean deliveries out of 99 in the insulin therapy group and 26 cesarean deliveries out of 97 in the metformin group, with a risk difference of 0.25 (95% CI 0.11, 0.38), favoring the use of metformin to prevent cesarean delivery. The guidelines from the Pan American Health Organization (PAHO), along with the Ministry of Health, the Brazilian Federation of Gynecology and Obstetrics Associations (FEBRASGO), and the Brazilian Diabetes Society advocate that, regardless of the pharmacotherapy used in the treatment of GDM, the outcome that most impacts the decision of the delivery route is fetal macrosomia, closely associated with shoulder dystocia and its complications.^(8,26–28)^

In conclusion, the indication of the delivery route is fundamentally obstetrical and should be individualized according to the estimated fetal weight on ultrasound, clinical evaluation of the woman, and careful assessment of fetal well-being, regardless of the pharmacotherapy used in the treatment of GDM.

Regarding the outcome of the need for admission to the NICU, only one selected article showed a statistically significant difference, Ainuddin et al.,^(18)^ which demonstrated a lower number of neonates requiring NICU in the group that used metformin. 11 newborns required NICU out of 50 in the insulin group, and 7 out of 47 in the metformin group, with a risk difference of 0.19 (95% CI 0.03, 0.36). This finding corroborates the results found in the systematic review conducted in 2023 by Sheng et al.,^(25)^ which analyzed 18 articles, with a total of 3527 neonates. The proposed meta-analysis shows lower chances of needing NICU with metformin use compared to insulin use (RR 0.73; 95% CI 0.61, 0.88; I^2^ = 23%; p = 0.0009).^(18,25)^

The rates of NICU admission are related to various factors such as premature birth, hypoglycemia, respiratory distress syndrome, and neonatal jaundice. In the aforementioned meta-analysis, the offspring of mothers treated with insulin required additional treatment for hypoglycemia, which is associated with an increase in NICU admissions.^(25)^

Therefore, it is concluded that although studies indicate a lower need for NICU admission in neonates of mothers exposed to metformin compared to insulin therapy for the treatment of GDM, its evidence was not clear since only one of the included studies showed a statistically significant difference.

Regarding the gestational age at birth outcome, no article showed a statistically significant difference between metformin and insulin in the presence of GDM. However, in the 2019 review by Bao et al.,^(29)^ involving seven studies with 847 GDM patients, a significant difference between the two groups was observed (RR -0.29; 95% CI -0.46, -0.11; I^2^ = 0%; p = .00). It is suggested, therefore, that metformin may shorten pregnancy and induce premature delivery.^(29)^

The American College of Obstetricians and Gynecologists (ACOG) suggests birth between 39+0 and 39+6 weeks of gestation for patients with well-controlled GDM with medication. However, guidance for patients with suboptimal glycemic control on pharmacological therapy is less precise. It is understood that birth between 37+0 and 38+6 weeks of gestation may be reasonable, but delivery before 37+0 weeks should only be initiated when more aggressive efforts to control blood glucose levels, such as hospitalization, fail.^(30)^

It is clear, therefore, the need for new studies for a more accurate assessment of the impact of metformin on the outcome of gestational age at birth.

When evaluating the outcome of neonatal hypoglycemia (NH), it was observed that two selected articles showed statistically significant differences: Picón-César et al.,^(8)^ and Ainuddin et al.^(18)^ Both demonstrated that pregnant women who used metformin had a lower risk of the neonate developing NH.^(8,18)^

In the study by Ainuddin et al.,^(18)^ the risk difference was 0.17 (95% CI 0.05, 0.28) in favor of using metformin to reduce the risk of NH. Indeed, in the insulin therapy group, 16 out of 75 neonates experienced this outcome, compared to only 2 out of 43 neonates in the metformin group. In the study by Picón-César et al.,^(8)^ 21 out of 99 newborns in the insulin therapy group and 6 out of 97 in the metformin group developed NH, with a risk difference of 0.15 (95% CI 0.06, 0.24) in favor of metformin. This finding corroborates with the systematic review and meta-analysis conducted by Sheng et al.,^(25)^ a study already cited previously. In the NH analysis, 20 articles involving 3670 neonates were included. The study showed that metformin decreased the incidence of NH with a risk difference of 0.65 (95% CI 0.52, 0.81; p = 0.0001).^(8,18,25)^

However, caution is needed when stating that metformin reduces the risk of NH, as only 2 out of the 7 articles evaluated in this study showed statistically significant differences. Thus, it is concluded that new studies are needed to ensure the benefit of metformin in the outcome of neonatal hypoglycemia.

Regarding the evolution towards preeclampsia, the only selected article that showed a statistically significant difference was Ainuddin et al.'s.^(18)^ The study indicated a lower risk of developing pre-eclampsia in patients undergoing metformin therapy compared to those using insulin. This finding is consistent with Yu et al.'s systematic review from 2021, which analyzed 30 articles and demonstrated a lower prevalence of pre-eclampsia in the metformin group (DR -0.03; 95% CI -0.06, 0, p = 0.02). Additionally, the study showed that metformin therapy had a higher likelihood of preventing cases of pre-eclampsia (87%) compared to insulin therapy (59%).^(18)^

However, despite the results found in the mentioned articles, there is no clear evidence of the benefit of metformin in this outcome, since most articles included in this study did not present results with statistically significant differences. Therefore, it is necessary for new studies to compare the incidence of this disease in the treatment of GDM with insulin therapy and metformin to ensure appropriate decision-making.

According to the analysis of the results of the studies conducted by Ruholamin et al.^(17)^ and Ghomian et al.,^(20)^ it is noted that there is no significant difference in the Apgar score ≤ 7 at the 5th minute of life between newborns of mothers treated with insulin compared to those treated with metformin. Therefore, it is important to conduct more studies that assess this outcome with greater care.^(17,20)^

Regarding the outcome of newborns large for gestational age (LGA) at birth, no selected study showed a statistically significant difference. However, the systematic review by Sheng et al., in 2023,^(25)^ analyzed 22 studies involving 4174 newborns and demonstrated that newborns whose mothers were treated with metformin had a 30% reduced risk of macrosomia compared to the insulin therapy group in 20 studies (RR 0.75; 95% CI 0.54, 0.86; I^2^ = 17%; p = 0.001).^(25)^

The main cause for fetuses being born LGA is related to GDM. Fetal macrosomia accounts for about 15-45% of newborns of diabetic mothers, a value three times higher than in nondiabetic mothers, and is dependent on glycemic control values. Additionally, the study conducted by Feig et al., in 2020^(31)^ demonstrated that lower neonatal adiposity in the metformin group led to a lower incidence of fetal macrosomia. Therefore, the action of metformin on macrosomia may be related to the inhibition of fetal fatty acid synthesis. It is worth noting that LGA fetuses present greater perinatal complications compared to appropriate for gestational age (AGA) newborns, such as a higher risk of meconium aspiration, clavicle fracture, shoulder dystocia, perinatal hypoxia, hypoglycemia, hyperbilirubinemia, transient tachypnea, brachial plexus injury, and neonatal death. Finally, despite no study in this systematic review showing statistical significance, the treatment of GDM with metformin appears to be safe and effective regarding fetal macrosomia and with better outcomes compared to insulin therapy. However, outcomes such as childhood weight gain should be evaluated and considered. There is a clear need for further studies addressing fetal macrosomia and, primarily, the ideal management in this clinical situation, aiming to address the knowledge gaps highlighted in this article.^(31)^

## Conclusion

We have demonstrated, through this study, the potential viability of using metformin as an alternative compared to insulin therapy in the treatment of Gestational Diabetes Mellitus. However, all assessed outcomes have a very low level of certainty of evidence. Further studies with longer follow-up periods, to assess maternal and neonatal outcomes in the medium and long term, are needed to add higher quality evidence and to better evaluate the use of metformin in GDM, since there was no standardization of medication doses and each study used different regimens of metformin and insulin therapy without direct comparison between the two medications.
